# Supplementary material for: Terahertz rectification in ring-shaped quantum barriers
Source: Nat Commun. 2018 Nov 21;9:4914. doi: 10.1038/s41467-018-07365-w (PMC6249216; doi:10.1038/s41467-018-07365-w)
Supplement: Supplementary file 1 — Supplementary Information [file 41467_2018_7365_MOESM1_ESM.pdf]

SUPPLEMENTARY INFORMATION

# Terahertz rectification in ring-shaped quantum barriers

Kang et al.

**Supplementary Note 1: Contour integration of barrier potentials**

We are interested in the summation of potentials over an arbitrarily shaped quantum barrier *C* under an external current density **K** (Fig. 1a). We assume that the surface current does not have any sources or sinks near the contour region (i.e. $\nabla \cdot \mathbf{K} = 0$). Contour integration of the electric potential $V(l,t)$ which is charged by the surface current can be written by,

$$\oint_C V(l,t)\,dl \propto \int_{-\infty}^{t} dt' \oint_C dl\,\mathbf{K}(l,t')\cdot\mathbf{n} \tag{1}$$

By using the vector relation $\mathbf{n}dl = d\mathbf{l} \times \hat{\mathbf{z}}$, the contour integral on the right hand side can be expressed by

$$\oint_C \mathbf{K}\cdot\mathbf{n}\,dl = \oint_C \mathbf{K}\cdot(d\mathbf{l}\times\hat{\mathbf{z}}) \tag{2}$$

Rearranging the integrand and using Stokes' theorem, Supplementary Eq. 2 can be written by,

$$\oint_C (\hat{\mathbf{z}}\times\mathbf{K})\cdot d\mathbf{l} = \iint_S \nabla\times(\hat{\mathbf{z}}\times\mathbf{K})\cdot d\mathbf{a} \tag{3}$$

where the operation on *S*, surface enclosed by the contour *C*, is performed in the *x*-*y* plane. The integrand of the surface integral can be expanded as follows,

$$\nabla\times(\hat{\mathbf{z}}\times\mathbf{K}) = \hat{\mathbf{z}}(\nabla\cdot\mathbf{K}) - (\hat{\mathbf{z}}\cdot\nabla)\mathbf{K} \tag{4}$$

The first term of right hand side vanishes by the assumption and the second term also vanishes since the surface current does not depend on *z*. Therefore, the value of the integration in Supplementary Eq. 1 vanishes independent of the current density **K** and the shape of the contour *C*, indicating that the total current resulting from Ohm's law across the closed barrier is zero; the closed-loop geometry automatically eliminates linear current components of Ohmic nature.

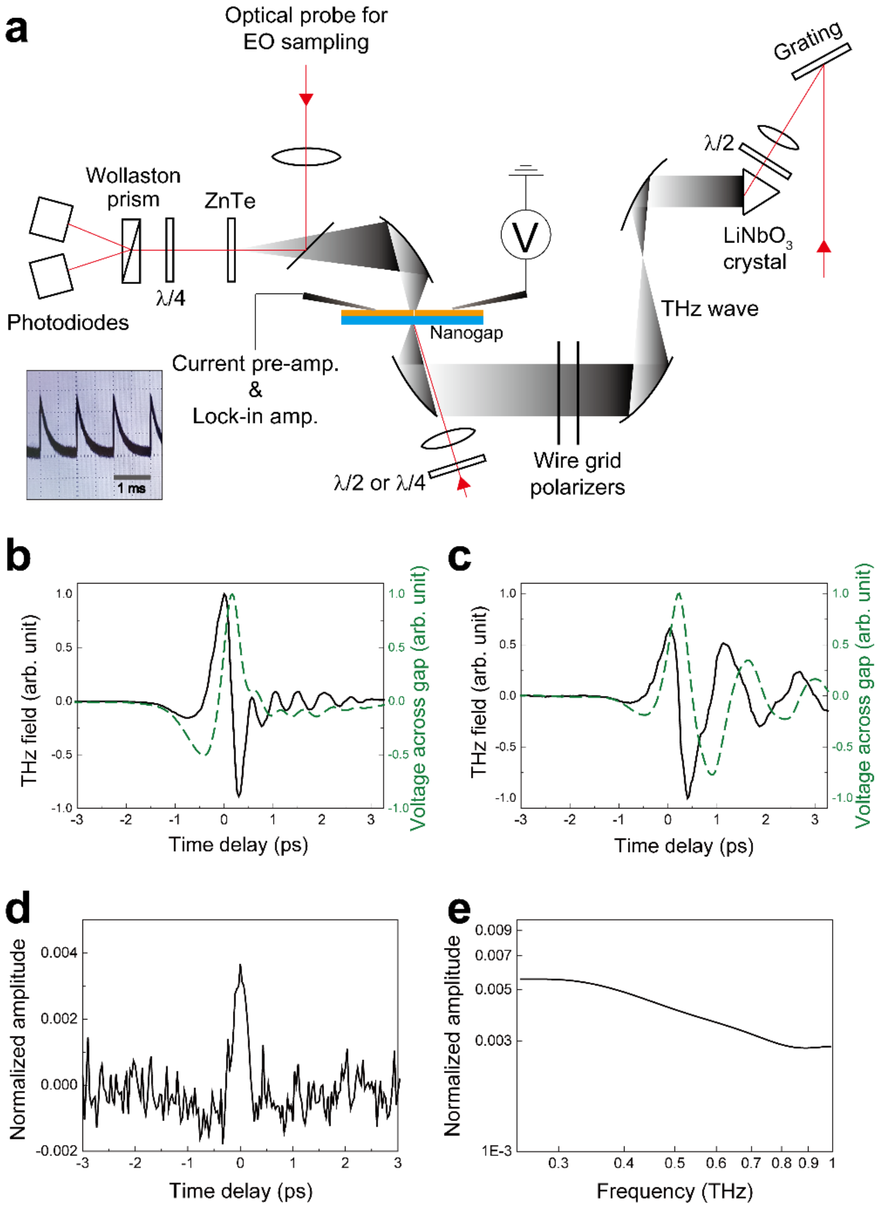


**Supplementary Figure 1 | Terahertz time-domain spectroscopy and tunneling current measurement.** (**a**) Schematic of the experimental setup for THz time-domain spectroscopy and tunneling current measurements. Inset shows an oscilloscope waveform of tunneling currents from the preamplifier. Current bursts are repeated at the THz pulse rate and affected by the bandwidth of the current preamplifier. (**b**) Generated single-cycled THz electric field time profile and its voltage profile by using Eq. 1 of the main manuscript. (**c**) Generated multi-cycled THz electric field time profile and its voltage profile by using Eq. 1 of the main manuscript. A 0.68 THz spectral filter was exploited to generate the multi-cycled pulse. (**d**) Measured transmitted amplitude of triangle nanogaps (side length of 70 μm, pattern period of 140 μm and gap size of 2 nm) in time-domain and **(e)** frequency-domain.

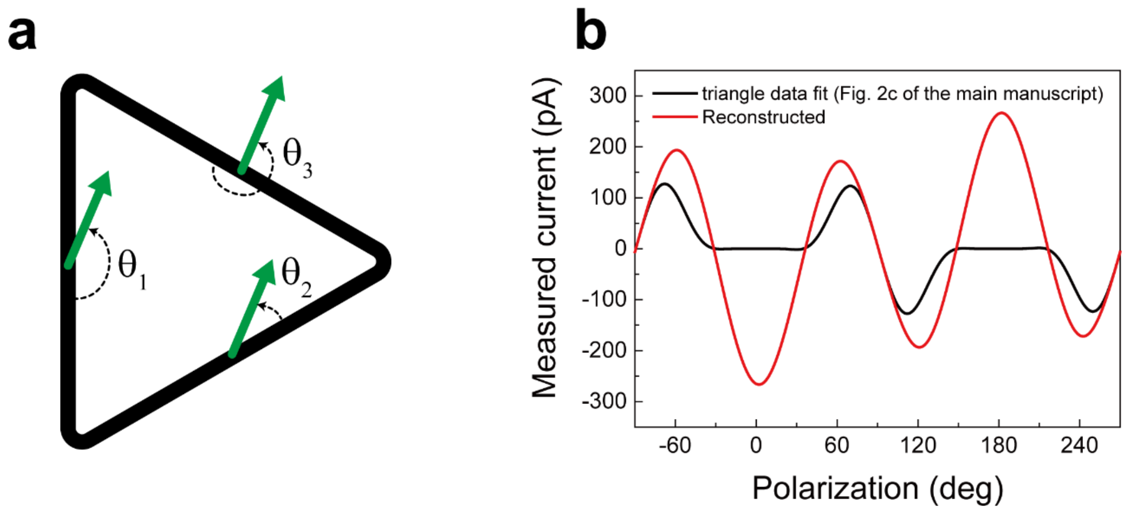


**Supplementary Figure 2 | Modeling polarization response of a triangular barrier.** (**a**) Schematic description of angles formed between a surface current induced by a THz pulse and sides of a triangle. The surface current applies potential across the gaps depending on the angles, thus the total tunneling current direction is sensitive to the polarization. (**b**) Polarization-dependent tunneling current of a triangle barrier. The black line is a fitting to the data presented in Fig. 2c based on the Simmons formula. By including the effect of polarizer rotation, we reconstructed the full 360 degree polarization rotation current, and presented as a polar plot in Fig. 2d (see Methods for details).

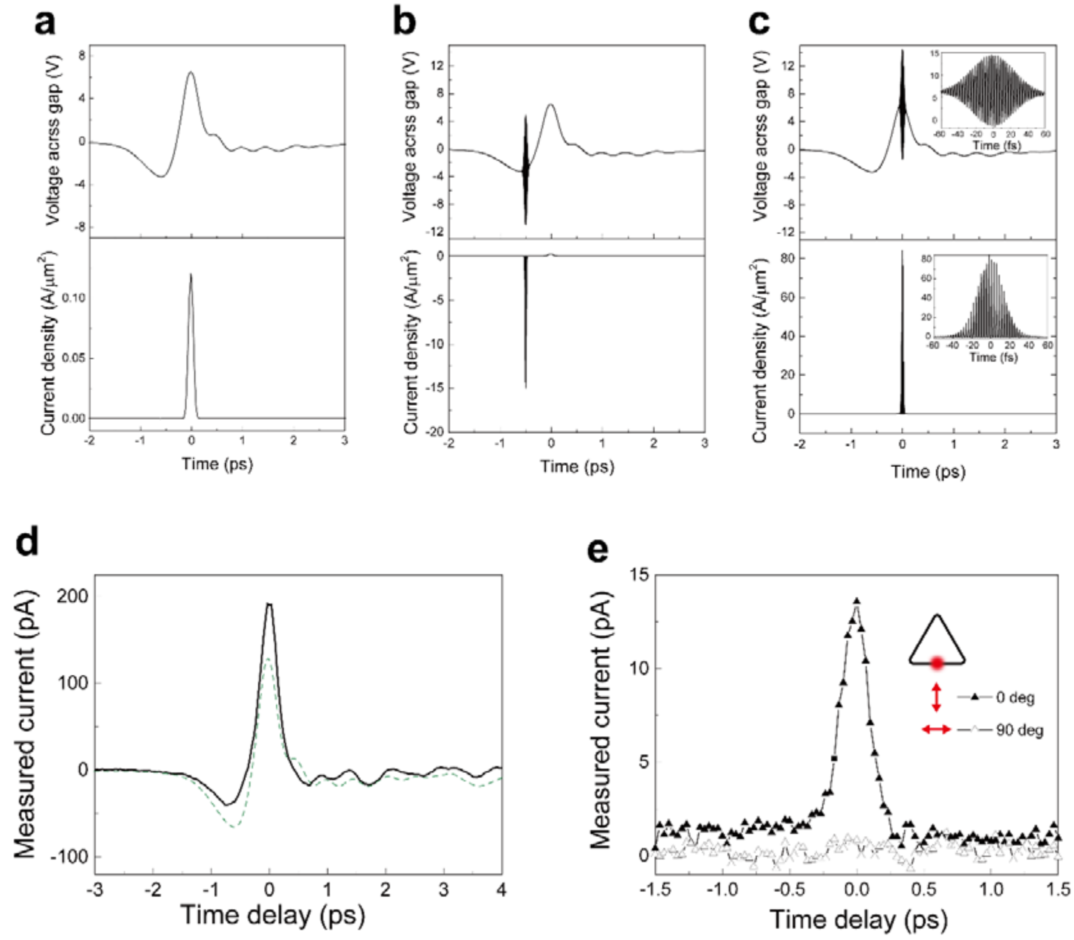


**Supplementary Figure 3 | Modeling optical currents.** Voltage time profile and its tunneling current transient calculation across a single junction when (**a**) only THz field is applied, (**b**) optical field is added at a delay of - 0.5 ps and (**c**) at zero-delay. Here the THz voltage is calculated by using the incident field $H_{inc}$ and Eq. 1 of the main manuscript (see Supplementary Fig. 1b). Most of the tunneling current is triggered only near the maximum of the voltage across gap. The optical pulse is assumed to be a sinusoidal profile with a Gaussian envelope of ~ 40 fs duration. Insets of (**c**) are magnification near the zero delay. (**d**) Measured optical tunneling current under the THz field illumination by changing optical delay (triangle barrier, side length of 500 μm, gap size of 2 nm). Green dashed line is a guide to the eye, representing the THz voltage profile which is the same curve shown at the top of (**a**). The optical method does not severely distort or change the order of the strength of currents driven by THz fields; hence, it can be used to demonstrate time-resolved THz current dynamics through the quantum barriers. **(e)** Measured optical responses of THz tunneling currents by changing the optical polarization. 0 degree means the optical polarization vertical to the side, and 90 degrees parallel to the side. The angle between the contour element and optical surface current direction also critically affects to the optical tunneling process, the same as for the THz tunneling case.

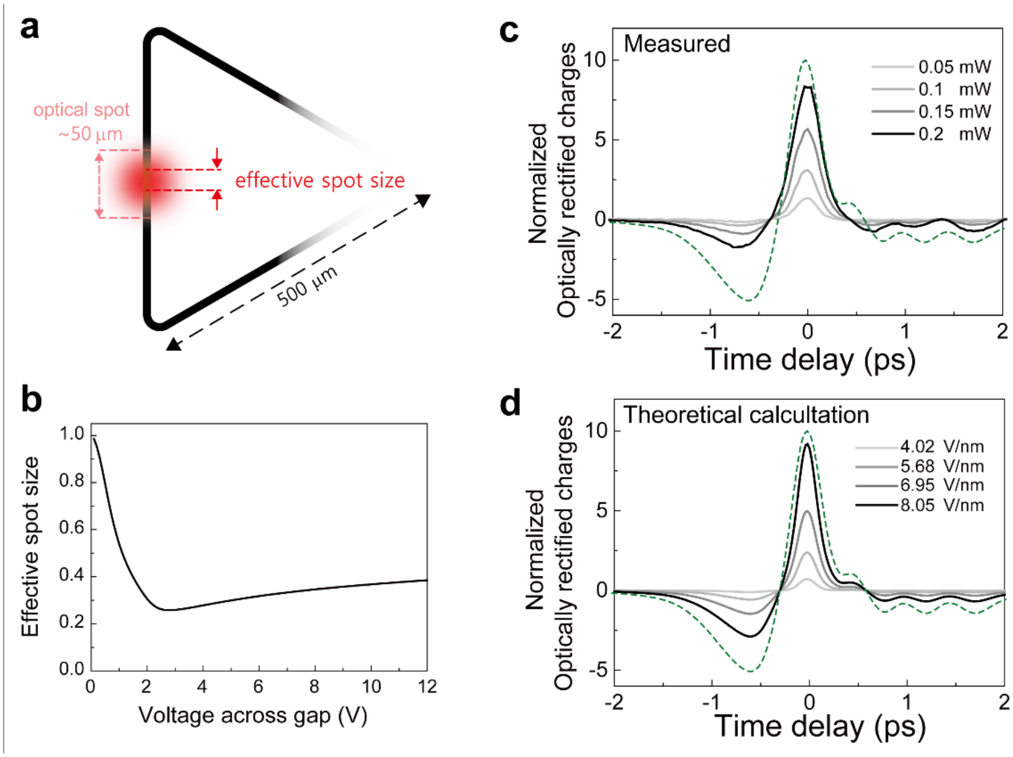


**Supplementary Figure 4 | Quantification of optically rectified charges.** (**a**) Schematic description for a quantitative modeling of optical tunneling currents under a quasi-static THz field. A 500 μm-sized triangle (gap size of 2 nm) is used to quantify tunneling currents through a single-sided barrier. Here, we assumed an equally distributed THz field (~ 1 mm spot size) strength along the side and ignored the other adjacent sides. Tunneling nonlinearity is taken into account for calculating the effective area for an optically-driven current flow. An effective optical spot size, defined by the length of the barrier normalized to the original optical spot size (~ 50 μm), where the optical current flows dominantly, is thus smaller than the original optical spot size. Both the THz field and optical field are incident with polarization of $\varphi_{pol} = 180^{o}$. (**b**) Calculated effective spot size as a function of the optical potential across the barrier. Effective spot size of 1 indicates the original optical spot size. (**c**) Measured and (**d**) calculated optically rectified charges as a function of the time delay. Here, the normalized-optically-rectified-charges is defined by $(q_{THz+opt}(t) - q_{THz})/q_{THz}$, where $q_{THz+opt}(t)$ is the rectified charges with the THz and optical field illumination as a function of delay $t$, and $q_{THz}$ is the rectified charges only with the THz field illumination. Legend in (**c**) indicates incident optical powers used in measurement and legend in (**d**) shows optical field strengths in the gap used for the calculation (optical field enhancement of 5.8[1] was used). Green dashed line indicates the THz voltage profile across the gap.

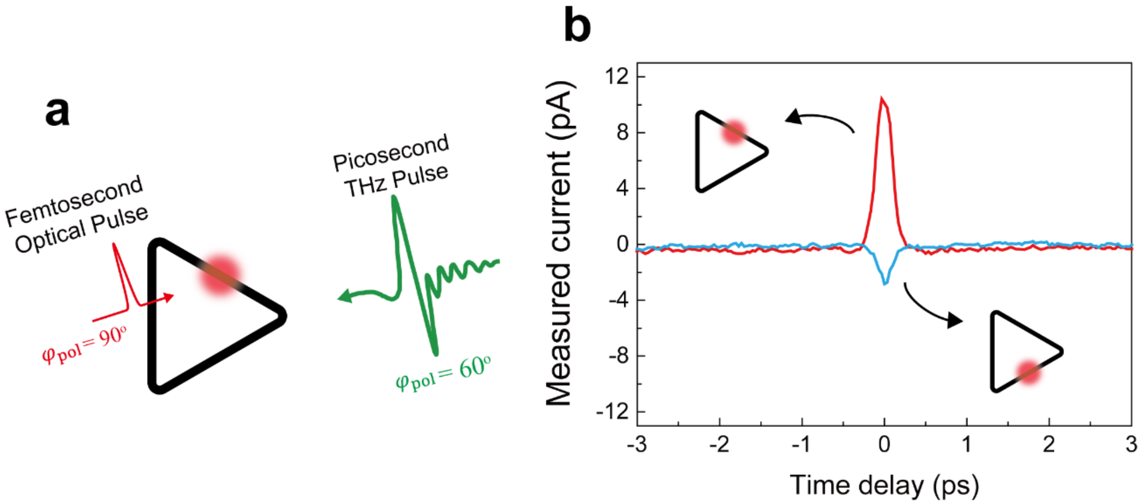


**Supplementary Figure 5 | Optical manipulation of the THz tunneling current II.** (**a**) Ultrafast THz tunneling currents are manipulated by an optical pulse illumination on the ring shaped quantum barriers. Femtosecond optical pulse generates an additional tunneling current at a specific position of the triangle. (**b**) Experimental demonstration of the ultrafast optical gating of the THz tunneling current is shown as a function of position and time delay of the optical pulse (triangle loop, side length of 70 μm, gap size of 2 nm). The optical pulse (focused down to a spot size of 50 μm) modulates the barrier potential spatiotemporally, thereby generating position-sensitive and time-dependent switching signals. Incident polarizations of THz and optical pulse are the same as in (**a**).

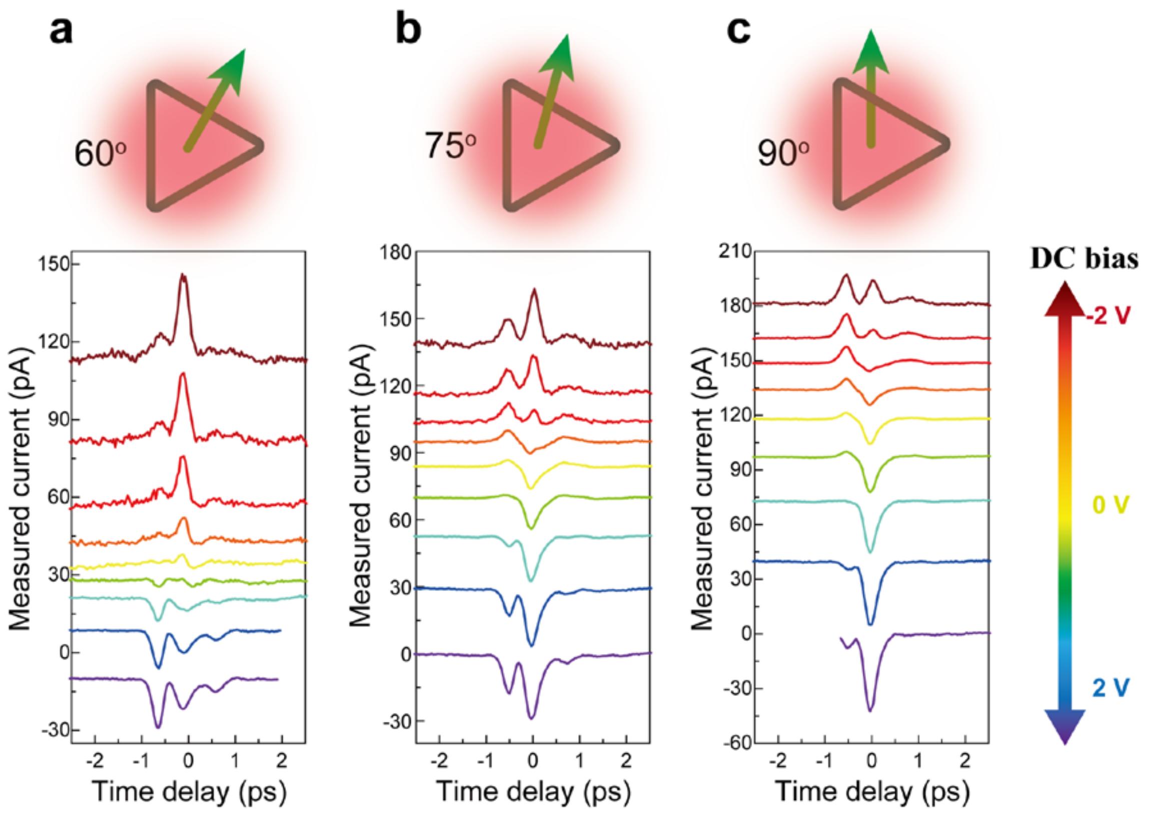


**Supplementary Figure 6 | Manipulation of THz rectifications by incident polarizations.** Pulse shaping of a fully rectified total THz tunneling current through the triangle barrier (side length of 100 μm, gap size of 4 nm) by changing THz polarization of (**a**) $\varphi_{pol}$ = 60º, (**b**) $\varphi_{pol}$ = 75º, and (**c**) $\varphi_{pol}$ = 90º, visualized by circularly polarized optical pulse which is expanded to cover the entire loop. The DC bias was varied from – 2 V to 2 V with 0.5 V step (right color bar).

**Supplementary References**


1 Ahn, J. S. *et al.* Optical field enhancement of nanometer-sized gaps at near-infrared frequencies. *Opt. Express* **23**, 4897-4907, doi:10.1364/OE.23.004897 (2015).
